# Risk Factors for Neonatal Sepsis in Pregnant Women with Premature Rupture of the Membrane

**DOI:** 10.1155/2018/4823404

**Published:** 2018-10-01

**Authors:** Dwiana Ocviyanti, William Timotius Wahono

**Affiliations:** Department of Obstetrics and Gynecology, Faculty of Medicine, Universitas Indonesia/Cipto Mangunkusumo Hospital, Jakarta, Indonesia

## Abstract

**Background:**

Premature rupture of the membrane (PROM) is associated with high maternal as well as perinatal morbidity and mortality risks. It occurs in 5 to 10% of all pregnancy while incidence of amniotic membrane infection varies from 6 to 10%. This study aimed to determine the incidence of neonatal sepsis in Cipto Mangunkusumo Hospital and the risk factors.

**Methods:**

A cross-sectional study was done in Cipto Mangunkusumo Hospital, Jakarta, from December 2016 to June 2017. The study used total sampling method including all pregnant women with gestational age of 20 weeks or more experiencing PROM, who came to the hospital at that time. Samples with existing comorbidities such as diabetes mellitus or other serious systemic illnesses such as heart disease or autoimmune condition were excluded from the analysis.

**Results:**

A total of 405 pregnant women with PROM were included in this study. There were 21 cases (5.2%) of neonatal sepsis. The analysis showed that risk of neonatal sepsis was higher in pregnant women with prolonged rupture of membrane for ≥ 18 hours before hospital admission (OR 3.08), prolonged rupture of membrane for ≥ 15 hours during hospitalization (OR 7.32), and prolonged rupture of membrane for ≥ 48 hours until birth (OR 5.77). The risk of neonatal sepsis was higher in preterm pregnancy with gestational age of <37 weeks (OR 18.59).

**Conclusion:**

Risk of neonatal sepsis is higher in longer duration of prolonged rupture of membrane as well as preterm pregnancy.

## 1. Introduction

Premature rupture of membrane (PROM) is the rupture of the amniotic membrane before the onset of labor [1]. PROM is associated with high maternal as well as perinatal morbidity and mortality risks [2]. It occurs in 5 to 10% of all pregnancy and 8 to 10% of term pregnancy. Amniotic membrane infection is one of pregnancy complications that may occur in pregnancy with PROM, in both preterm and term pregnancies. In term pregnancy, the incidence of amniotic membrane infection varies from 6 to 10% and occurs in 40% of prolonged PROM that persists for more than 24 hours [3]. In preterm pregnancy, preterm premature rupture of the membrane occurs in 2.0% to 3.5% of pregnancies and is the most common cause of preterm birth, present in 30% to 40% of cases [4]. Sequelae of amniotic membrane infection are potentially fatal in pregnant women and their babies [3].

In 2005, the WHO reported that 37% of child mortality occurs below 5 years of age, and neonatal sepsis accounted for 29% of deaths within that age group [5]. Results of an epidemiological study done by the WHO and UNICEF in 2010 found that there were 7.6 million cases of under-five mortality, in which 64% (4.879 million) occurred due to infection and the remaining 40.3% (3.072 million) occurred in neonates [6]. The latest SDKI report in 2012 showed that Infant Mortality Rate (IMR) in Indonesia is at 32/1,000 live births [7]. Sepsis or meningitis is one of the leading causes of neonatal death, accounted for 5.2% (0.393 million) [6].

The incidence of neonatal infection after rupture of membrane that persists for more than 24 hours is 1%, and after clinical inspection, the incidence mounts up to 3-5%. In general, tenfold increase in neonatal infection occurred in premature rupture of membrane cases without complications [8]. A multicenter study on PROM in term pregnancy, conducted in the US, Canada, UK, and Israel, found that prolonged rupture of membrane for ≥48 hours and 24 to 48 hours increases the risk of neonatal infection by 2.25 times [3]. Some studies on preterm PROM showed no association between prolonged rupture of membrane with neonatal infection. However, a meta-analysis study found significant association between antibiotic administration in mothers with incidence of neonatal infection (OR 0.68 [0.53-0.87]) [9–12]. Based on the previous data, we decide to perform this study to find out the true incidence of neonatal sepsis and risk factors related to them.

## 2. Materials and Methods

This study is a hospital-based analytical descriptive study done in Cipto Mangunkusumo Hospital, Jakarta, for 7 months, since December 2016 until June 2017. The study used total sampling method, in which all pregnant women with PROM and gestational age of more than 20 weeks admitted since 1st January to 31st December of 2016, as well as their babies, are included. In terms of maternal data, we collect age, level of education, working status, parity, gestational age, mode of delivery, and mother hemoglobin. In terms of neonatal data, we collect birthweight, length of stay, APGAR score, treatment with antibiotics, and neonatal death. Subjects with existing comorbidities and complications such as diabetes mellitus, intrauterine infection, and other serious systemic illnesses, e.g., lung and heart diseases, autoimmune conditions, fetal congenital abnormality, and multiple pregnancy, were excluded. Subjects with incomplete medical record were also excluded. Data was analyzed using Stata 12.

## 3. Results

There were 488 cases of pregnant women with PROM in Cipto Mangunkusumo Hospital throughout the year of 2016. Of that number, a total of 405 women met the inclusion criteria. The remaining 83 were excluded.

Of 405 PROM cases, 21 (5.2%) suffered from neonatal sepsis. Of all PROM cases, 186 (45.9%) occurred in term pregnancy, of which 56 cases (30.1%) were suspected neonatal sepsis and 130 cases (68.9%) were without neonatal sepsis. Of 56 cases with suspected neonatal sepsis, positive blood culture was found in only one case and alive. The other 55 cases showed negative blood cultures. Of this number, one died (1.8%) and the remaining 54 cases (98.2%) were alive.

PROM cases in preterm pregnancy occurred in 219 subjects (54.1%), of which neonatal sepsis was suspected in 128 cases (58.4%), and there was no sepsis in 91 cases (41.6%). Of 128 cases with suspected neonatal sepsis, 20 cases (15.6%) showed positive blood cultures. Of these, 8 neonates died (40%) and 12 were alive (60%). Meanwhile, of 108 cases with negative blood cultures, 97 neonates were alive (89.8%) and 11 died (10.2%). In cases without neonatal sepsis, neonatal death was found in 2 cases (2.2%) and the remaining 89 were alive (97.8%). The characteristics of subjects were presented on Table 1.

Neonatal sepsis occurred in only one subject with gestational age of ≥ 37 weeks (0.5%), compared to 20 subjects (9.1%) with gestational age of < 37 weeks. In preterm pregnancy with gestational age of 34 to less than 37 weeks, neonatal sepsis was found in 2 subjects (2.4%), while, in gestational age of 28 to less than 34 weeks, it was found in 13 subjects (10.8%). In gestational age of less than 28 weeks, neonatal sepsis was found in 5 subjects (29.4%). The distribution of risk factors based on neonatal sepsis incidence is presented in Table 2.

The average birth weight of babies with neonatal sepsis was 1,420 grams, compared to 2,560 grams in babies without neonatal sepsis. Babies with neonatal sepsis were hospitalized for 32 days on average, compared to 3 days in babies without the condition. Antibiotics were administered in all cases of neonatal sepsis (100%), whereas in those without the condition, 109 neonates (28.4%) were given. Administered antibiotics were ampicillin-sulbactam dan gentamycin, for duration of 10-14 days.

Neonatal death occurred in 8 cases (38.1%) with neonatal sepsis compared to 14 cases (3.7%) without the condition. The distribution of neonatal outcomes in pregnant women with PROM was presented in Table 3.

**Figure 1:**
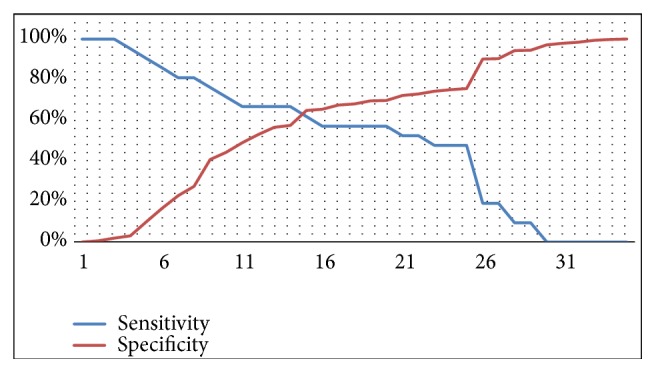
Cut-off point of duration from PROM to hospital admission in all PROM cases (18 hours).

**Figure 2:**
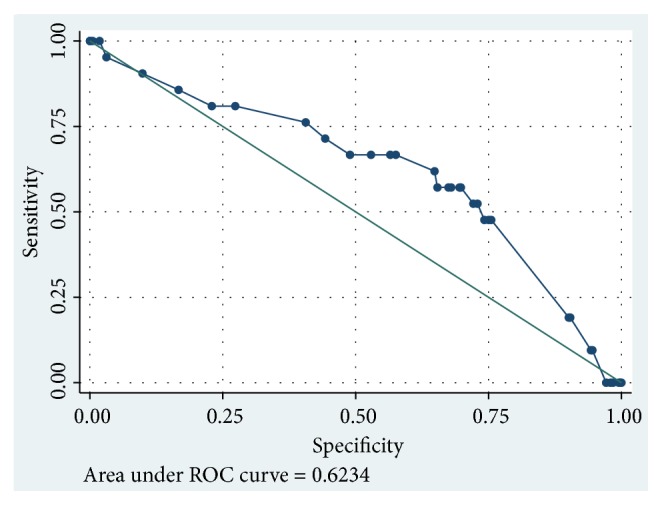
ROC curve duration from PROM to hospital admission in all PROM cases.

To determine the effect of prolonged rupture of membrane upon neonatal sepsis incidence, Youden index was used to identify the cut-off point of Receiver Operating Characteristics (ROC) curve index. Cut-off point with the most optimum sensitivity and specificity value was then selected. Because of this, the cut-off point appeared on the graphs and tables were slightly different. In all PROM cases, cut-off point was 18 hours (Sn 60%, Sp 66.33%), 15 hours (Sn 80.95%, 63.28%), and 48 hours (Sn 66.67%, Sp 76.56%) for prolonged rupture of membrane before hospital admission, during hospitalization, and until birth, respectively. Cut-off point of duration from PROM to hospital admission was illustrated in Figure 1 and ROC curve of duration from PROM to hospital admission was illustrated in Figure 2.

ROC curve for the number of vaginal examination variable can not be identified. Thus, no analysis was performed. Based on neonatal sepsis incidence, the distribution of vaginal examination was as follows: 0 in 14 cases, 1 time in 5 cases, 2 times in 1 case, and 9 times in 1 case.

To identify the association between prolonged rupture of membrane as well as gestational age with the incidence of neonatal sepsis, bivariate analysis was performed. Statistically significant association (p<0.05) was found in prolonged rupture of membrane before hospital admission (OR 3.08), during hospitalization (OR 7.32), and until birth (OR 5.77). Gestational age also showed statistically significant association (p<0.05) with OR 2.9. The association of these variables with neonatal sepsis incidence can be found in Table 4.

Subanalysis was performed on PROM cases occurring in preterm pregnancy. Youden index was again used to determine the cut-off point of ROC curve. In preterm pregnancy, the cut-off point on prolonged rupture of membrane was 18 hours, 38 hours, and 59 hours, respectively, before hospital admission, during hospitalization, and until birth.

Bivariate analysis of this subanalysis also showed significant association (p<0.05) between prolonged rupture of membrane before hospital admission (OR 2.95), during hospitalization (OR 4.03), and until birth (5.69) with neonatal sepsis incidence. The association of these variables with neonatal sepsis incidence in preterm pregnancy of less than 37 weeks can be found in Table 5.

## 4. Discussion

Our study shows that neonatal sepsis incidence proven by positive blood cultures was 5,2%. This number is slightly higher compared to study conducted by Van Der Ham et al. in 2014 where neonatal sepsis occurred in 3.4% of all PROM cases. Similar study by Popowski et al. showed neonatal sepsis incidence of 4.3% [13, 14]. Based on the 2015 report of Department of Child Health at Cipto Mangunkusumo Hospital, neonatal sepsis incidence in the hospital was 13.01% [15]. In contrast, studies from several local referral hospital showed that neonatal incidence in Indonesia varied between 1.5% and 3.7% [16].

This study showed that more than 50% of subjects with PROM had preterm pregnancy. This is quite reasonable since Cipto Mangunkusumo is a national referral hospital which provides NICU. In the hospital, delivery method was mostly done by caesarean section in most patients with PROM (75.3%). This number is higher than the study conducted by Pasquier et al. that was 58% [9]. Based on local guidelines at Cipto Mangunkusumo Hospital, for PROM cases in preterm pregnancy, after lung maturation patient without any immediate medical indication (such as pathological CTG) would be given informed choice to decide whether to have C-section or continue with induction of labor (with consequences of a longer duration of PROM due to induction). This might be the cause of high rate of caesarean section in PROM cases, because patient chose to have caesarean section to avoid further risk of neonatal infection.

However, caesarean section on maternal request is still a debatable topic. A study conducted by Indraccolo et al. in Italy found that OBGYNs and midwives agree that performing a planned caesarean section on maternal request (CSMR) without medical indication being considered an error. However, from lawyers point of view, following patient's own decision to have CSMR, independently from medical complication, is considered pivotal. The study also found that if patients ask for a caesarean but the OBGYN does not perform a planned CSMR, patients feel that the physician's decision in case of a vaginal delivery complication is juridically relevant, and it appears that patients would be more likely to lodge a claim in case of complications if the OBGYN does not perform a CSMR [17]. It is very important to develop a prediction model to predict neonatal sepsis in cases of PROM, which can help both the patient and clinician to decide which is best for maternal and neonatal outcome.

Regarding neonatal outcomes related to neonatal sepsis incidence, it was found that the average birth weight of babies suffering from neonatal sepsis was 1,420 grams, while for those without neonatal sepsis it was 2,560 grams. Since 95% of neonatal sepsis occurred in preterm pregnancy, low birth weight of babies was expected. Babies with neonatal sepsis also had longer length of stay in the hospital, with median of 32 days, compared to 3 days in those without the condition. This finding might as well be related to the preterm gestational age. It is also consistent with the study by Manuck et al., where duration of hospitalization is longer in preterm neonates [18].

Based on gestational age of mothers, neonatal sepsis in terms of pregnancy (≥ 37 weeks) was found in only 1 subject (0.5%) compared to 20 subjects (9.1%) in preterm pregnancy. It implies that prematurity is an important factor on the occurrence of neonatal sepsis. Manuck et al. also stated that preterm pregnancy of less than 37 weeks is the most frequent cause of neonatal morbidity [18]. Results from bivariate analysis showed that gestational age was significantly associated with neonatal sepsis incidence (p<0.05). The OR for neonatal sepsis in preterm pregnancy was 18.59. When we tried to further divide preterm gestational age into 3 groups, i.e., <37 weeks, 34 to <37 weeks, and 28 to <34 weeks, an increasing trend of neonatal sepsis risk was observed in more preterm pregnancy. The odds are 4.63, 22.48, and 77.08, respectively. Similar trend was also found by Manuck et al., in which trends of neonatal morbidity and infection were higher at younger gestational age [18].

In all PROM cases, regardless of gestational age, bivariate analysis showed significant association (p<0.05) between prolonged rupture of membrane before hospital admission, during hospitalization, and until birth with neonatal sepsis incidence. The odds ratio was 3.09, 7.32, and 5.77, respectively.

Subanalysis was then performed for PROM cases in preterm pregnancy of less than 37 weeks. It was again found that there was significant association (p<0.05) between prolonged rupture of membrane before hospital admission, during hospitalization, and until birth with neonatal sepsis incidence. The odds ratio was 2.95, 4.03, and 5.69, respectively.

These findings showed that the longer the duration of membrane rupture, the higher the risk of neonatal sepsis. However, different results were shown by Drassinower et al., where prolonged rupture of membrane of ≥ 4 weeks was associated with lower incidence of neonatal sepsis. In this study, PROM with latent period of < 4 weeks occurred in younger gestational age (25.6 weeks). Meanwhile, PROM with latent period of ≥ 4 weeks occurred in older gestational age (28 weeks) where the birth weight was also greater [19].

## 5. Conclusion

Our study shows that risk of neonatal sepsis is higher in longer duration of prolonged rupture of membrane as well as preterm pregnancy.

## Figures and Tables

**Table 1:** Subjects characteristics of patient with PROM.

**Characteristic (n=405)**	**n (**%**)**
Maternal age	
< 20 yo	34 (8.4)
20 – 35 yo	308 (76.0)
> 35 yo	63 (15.6)
Level of education	
≤ 6 year	13 (3.2)
> 6 - ≤ 12 year	100 (24.7)
> 12 year	35 (8.6)
No data available	257 (63.5)
Working status	
Working	119 (29.4)
Not working / Housewife	249 (61.5)
No data available	37 (9.1)
Parity	
Multiparity	239 (59.0)
Nulliparity	166 (41.0)
Gestational age (2 categories)	
Preterm < 37 weeks	219 (54.1)
Aterm ≥ 37 weeks	186 (45.9)
Gestational age (4 categories)	
< 28 weeks	17 (4.2)
28 - < 34 weeks	120 (29.6)
34 - < 37 weeks	82 (20.3)
≥ 37 weeks	186 (45.9)
Mode of delivery	
Cesarean section	305 (75.3)
Vaginal delivery	100 (24.7)
Mother hemoglobin	
< 7 g/dL	4 (1.0)
7 – 11 g/dL	135 (33.3)
> 11 g/dL	266 (65.7)

Data are expressed in n(%) and mean ± standard deviation or median (min-max).

**Table 2:** Distribution of risk factors based on incidence of neonatal sepsis.

**Variable**	**With neonatal sepsis ** **n = 21 (5.2)**	**Without neonatal sepsis ** **n = 384 (94.8)**
Gestational age		
≥ 37 weeks	1 (0.5)	185 (99.5)
< 37 weeks	20 (9.1)	199 (90.9)
34 - < 37 weeks	2 (2.4)	80 (97.6)
28 - < 34 weeks	13 (10.8)	107 (89.2)
< 28 weeks	5 (29.4)	12 (70.6)
Duration from PROM to hospital admission (hour)	20 (1-72)	9 (0-600)
< 18 hour	9 (3.2)	268 (96.8)
≥ 18 hour	12 (9.4)	116 (90.6)
Duration from PROM to delivery (hour)	63 (6-247)	26 (2-647)
Duration from PROM during hospitalization (hour)	43 (3-223)	10 (1-164)

Data are expressed in n(%) and mean ± standard deviation or median (min-max).

**Table 3:** Distribution of neonatal outcome in pregnant women with PROM.

**Variable**	**With neonatal sepsis ** **n = 21 (5.2)**	**Without neonatal sepsis ** **n = 384 (94.8)**
Birthweight	1,420 (±410.3)	2,560 (±688.2)
Length of stay (day)	32 (3-79)	3 (0-85)
APGAR score		
First minute		
0 - 7	14 (13)	94 (87)
8 - 10	7 (2.4)	290 (97.6)
Fifth minute		
0 – 7	4 (17.4)	19 (82.6)
8 – 10	17 (4.5)	365 (95.5)
Treatment with antibiotics to neonates	21 (100)	109 (28.4)
Neonatal death	8 (38.1)	14 (3.7)

Data are expressed in n(%) and mean ± standard deviation or median (min-max).

**Table 4:** Bivariate analysis of duration of premature rupture of membrane with neonatal sepsis in all PROM cases in RSCM, 2016.

	**Neonatal sepsis**		**P value**	**OR**	**95**%** CI**	
	**Positive ** **(n=21)**	**Negative (n=384)**			**Min**	**Max**
Duration from PROM to hospital admission						
≥ 18 hour	12 (9.4)	116 (90.6)	0.009	3.08	1.15	8.49
< 18 hour	9 (3.2)	268 (96.8)				
Duration from PROM during hospitalization						
≥ 15 hour	17 (10.8)	141 (89.2)	< 0.001	7.32	2.32	30.37
< 15 hour	4 (1.6)	243 (98.4)				
Duration from PROM to delivery						
≥ 48 hour	8 (17.8)	37 (82.2)	< 0.001	5.77	1.93	16.11
< 48 hour	13 (3.6)	347 (96.4)				
Gestational age (2 categories)						
Preterm < 37 weeks	20 (9.1)	199 (90.9)	<0.001	18.59	2.90	774.57
Aterm ≥ 37 weeks	1 (0.5)	185 (99.5)				
Gestational age (4 categories)						
< 28 weeks	5 (29.4)	12 (70.6)	<0.001	77.08	8.33	713.29
28 - <34 weeks	13 (10.8)	107 (89.2)	0.003	22.48	2.89	174.22
34 - <37 weeks	2 (2.4)	80 (97.6)	0.214	4.63	0.41	51.74
≥ 37 weeks	1 (0.5)	185 (99.5)				

**Table 5:** Bivariate analysis of duration of premature rupture of membrane with neonatal sepsis in preterm PROM <37 weeks in RSCM, 2016.

	**Neonatal sepsis**		**p value**	**OR**	**95**%** CI**	
	**Positive (n=20)**	**Negative (n=199)**			**Min**	**Max**
Duration from PROM to hospital admission						
≥ 18 hour	12 (15.2)	67 (84.8)	0.019	2.95	1.05	8.72
< 18 hour	8 (5.7)	132 (94.3)				
Duration from PROM during hospitalization						
≥ 38 hour	12 (18.2)	54 (81.8)	0.002	4.03	1.41	11.94
< 38 hour	8 (5.2)	145 (94.8)				
Duration from PROM to delivery						
≥ 59 hour	13 (21)	49 (79)	<0.001	5.69	1.96	17.67
< 59 hour	7 (4.5)	150 (95.5)				

## Data Availability

The [EXCEL] data used to support the findings of this study are available from the corresponding author upon request.
